# Bilateral sympathectomy protects left ventricle against post-infarction remodelling

**DOI:** 10.1186/1749-8090-10-S1-A68

**Published:** 2015-12-16

**Authors:** Fernando L Zanoni, Rafael Simas, Raphael G Silva, Luiz FP Moreira

**Affiliations:** 1 Department of Cardiopneumology, Heart Institute (InCor) - University of Sao Paulo Medical School, Sao Paulo, 05403-000, Brazil

## Background/Introduction

Sympathetic activity influences the post-infarction ventricular remodelling and the use of beta-blockers has shown beneficial effects on the left ventricle (LV) function and remodelling after myocardial infarction (MI). Nevertheless, its use has some limitations and may cause adverse effects in certain patients.

## Aims/Objectives

Evaluate the influence of thoracic sympathectomy on LV remodelling and function post MI in rats.

## Method

Male Wistar rats (300 ± 50g) were anesthetized and, after left thoracotomy, MI was induced by ligation of left anterior descending (LAD) coronary. Sham rats were submitted only to thoracotomy. One week thereafter, MI rats were randomly submitted to left (LS) or bilateral (BS) sympathectomy by chemical ablation of stellate ganglion. Eight weeks after LAD ligation, the ventricular function was evaluated by conductance microcatheter technique. Stroke work (SW), cardiac output (CO), systolic volume (SV), end-systolic volume (ESV), end-diastolic volume (EDV), ejection fraction (EF) and dP/dt max were determined at baseline and after stimulation with dobutamine. Infarct size was determined by epicardial and endocardial infarct arc lengths.

## Results

Eight weeks after LAD ligation, MI and LS rats presented increased EDV (214 ± 15 μL and 234 ± 39 μL, respectively), whereas BS group preserved EDV (133 ± 29 μL), similar to Sham rats (133 ± 10 μL) (p = 0.002). A significant reduction in EF was observed in MI and LS groups (33 ± 5% and 35 ± 6%, respectively), whereas it was preserved in BS (51 ± 5%) and Sham rats (62 ± 3%) (p = 0.001). In response to dobutamine infusion, LV contractility increased in Sham rats, rising SW, CO, SV, EDV, EF, and dP/dt max. Although none infarcted groups did exhibit changes in EDV after dobutamine infusion, a significant increase in EF (p < 0.001) was observed in BS rats (77 ± 8%) in comparison to MI group (48 ± 10%). A slight increase in SW, CO and SV after dobutamine was observed in MI untreated rats and those submitted to sympathectomy. No differences was observed on infarct size among groups (p > 0.05).

## Discussion/Conclusion

Bilateral sympathectomy was effective to attenuate LV remodelling and to preserve systolic function after myocardial infarction induction in rats.

